# Cost-effectiveness of 5 fraction and partial breast radiotherapy for early breast cancer in the UK: model-based multi-trial analysis

**DOI:** 10.1007/s10549-022-06802-1

**Published:** 2022-11-17

**Authors:** David Glynn, Judith Bliss, Adrian Murray Brunt, Charlotte E. Coles, Duncan Wheatley, Joanne S. Haviland, Anna M. Kirby, Francesco Longo, Rita Faria, John R. Yarnold, Susan Griffin

**Affiliations:** 1 Centre for Health Economics, University of York, Heslington, UK; 2 Clinical Trials and Statistics Unit at The Institute of Cancer Research, London, UK; 3 School of Medicine, University of Keele, Staffordshire & Institute of Cancer Research, London, UK; 4 University of Cambridge, Cambridge University Hospitals NHS Foundation Trust, Cambridge, UK; 5 Royal Cornwall Hospital, Treliske, Truro, UK; 6 Royal Marsden NHS Foundation Trust & Institute of Cancer Research, Sutton, UK; 7 The Institute of Cancer Research: Royal Cancer Hospital, Sutton, UK

**Keywords:** Breast cancer, Radiotherapy, Economic evaluation, Hypofractionation, Partial breast

## Abstract

**Purpose:**

We estimated the cost-effectiveness of 4 radiotherapy modalities to treat early breast cancer in the UK. In a subgroup of patients eligible for all modalities, we compared whole-breast (WB) and partial breast (PB) radiotherapy delivered in either 15 (WB15F, PB15F) or 5 fractions (WB5F, PB5F). In a subgroup ineligible for PB radiotherapy, we compared WB15F to WB5F.

**Methods:**

We developed a Markov cohort model to simulate lifetime healthcare costs and quality-adjusted life years (QALYs) for each modality. This was informed by the clinical analysis of two non-inferiority trials (FAST Forward and IMPORT LOW) and supplemented with external literature. The primary analysis assumed that radiotherapy modality influences health only through its impact on locoregional recurrence and radiotherapy-related adverse events.

**Results:**

In the primary analysis, PB5F had the least cost and greatest expected QALYs. WB5F had the least cost and the greatest expected QALYs in those only eligible for WB radiotherapy. Applying a cost-effectiveness threshold of £15,000/QALY, there was a 62% chance that PB5F was the cost-effective alternative in the PB eligible group, and there was a 100% chance that WB5F was cost-effective in the subgroup ineligible for PB radiotherapy.

**Conclusions:**

Hypofractionation to 5 fractions and partial breast radiotherapy modalities offer potentially important benefits to the UK health system.

**Supplementary Information:**

The online version contains supplementary material available at 10.1007/s10549-022-06802-1.

## Introduction

Radiotherapy after primary surgery for people with early breast cancer has been shown to halve the risk of any breast cancer relapse at 10 years [1]. 2016 Guidance from the UK Royal College of Radiologists and 2018 guidance from the American Society for Radiation Oncology recommend no more than 15 fractions (15F) of whole-breast radiotherapy over three weeks for standard adjuvant treatment [2, 3]. 2009 clinical guidance from the National Institute for Health and Care Excellence (NICE), reaffirmed in updated 2018 guidance, recommended 40 Gy in 15F for women with invasive breast cancer after breast-conserving surgery or mastectomy [4, 5].

Two recent UK trials explored modifications to standard clinical practice. The FAST-Forward (FF) study compared a 40 Gy whole-breast radiotherapy dose delivered in 15F over three weeks to 27 Gy and 26 Gly delivered in five fractions (5F) over 1 week [6]. This trial found that 26 Gy 5F radiotherapy delivered over one week was non-inferior to 15F for the primary outcome of local (ipsilateral) relapse at 5 years following radiotherapy. Late normal tissue effects were similar after 26 Gy in 5F compared with 40 Gy in 15F. The IMPORT LOW (IL) study compared 40 Gy in 15F over three weeks delivered as whole or partial breast (PB) radiotherapy (and also included a “reduced dose” group, giving full dose to partial volume and reduced dose to the whole-breast volume) [7]. IMPORT Low used shortened tangential fields around the breast region containing the tumour bed. A homogenous dose was produced with simple intensity-modulated radiotherapy (IMRT). The justification for this approach was to minimise dose to important organs at risk (lungs and/or heart) and limit rare, but serious late cardio-pulmonary toxicity within a population at low risk of breast cancer relapse. At 5 years follow-up, IL demonstrated non-inferiority of PB radiotherapy with local relapse as the primary outcome. Late normal tissue effects were better or similar with PB radiotherapy due to the smaller irradiated volume.

Hypofractionated regimens have the potential to reduce treatment costs for the UK health system and reduce treatment burden for patients [6, 8]. PB radiotherapy reduces the exposure of internal organs and normal breast tissue to potentially harmful radiation and so is predicted to reduce long-term adverse events [7]. Taken together, these two studies indicate that both 5F therapy and PB radiotherapy may be useful in treating early breast cancer. These potential benefits have resulted in the UK Royal College of Radiologists providing a consensus statement in 2021 recommending offering 5F radiotherapy postoperatively in breast cancer delivered both as whole-breast and PB therapy[9]. However, the magnitude of potential benefits and cost savings is yet to be investigated formally.

The purpose of this paper is to formally evaluate the costs and health consequences associated with 5F and PB radiotherapy in the UK population. The cost-effectiveness analysis involved the development of a new decision model, to account for the various aspects of patient-centred value beyond the trials’ primary outcome of local relapse [10, 11], to consider the consequences on health outcomes and health service costs over the long-term beyond the trials’ follow-up and to predict the expected outcomes if PB radiotherapy is delivered in 5F over one week, which has not been evaluated in a trial [12, 13].

## Methods

### Target population

The target population was adults who have undergone breast-conserving surgery or mastectomy for early breast cancer (stage I/II/IIIa). Using the Royal College of Radiotherapists consensus statements 2016, we defined subgroups eligible for PB radiotherapy (subgroup 1) and ineligible for PB (subgroup 2) (see Table 1) [2]. The FF option of 27 Gy of whole-breast radiotherapy delivered in 5F and the IL mixed whole partial breast option were not evaluated here. This is because these arms were included in the trials for explanatory purposes and have not been taken up in clinical practice.

**Table 1:** Treatment options by subgroup

Population	Criteria	Treatment options
Subgroup 1: Eligible for partial breast radiotherapy	50 + years old, and tumour grade 1–2, and tumour size < = 3 cm, and estrogen receptor positive (ER +), and HER2 negative and have no regional lymph node metastasis (N0)	Whole breast 40 Gy in 15 Fractions (WB15F)
		Partial breast 40 Gy in 15 Fractions (PB15F)
		Whole breast 26 Gy in 5 Fractions (WB5F)
		Partial breast 26 Gy 5 Fractions (PB5F)
Subgroup 2: Not eligible for partial breast radiotherapy	Less than 50 years old, or tumour grade 3, or tumour size > 3 cm or estrogen receptor negative (ER-), or HER2 positive or with regional lymph node metastasis (N1 or greater)	Whole breast 40 Gy in 15 Fractions (WB15F)
		Whole breast 26 Gy 5 Fractions (WB5F)

### Decision analytic model

**Fig. 1:**
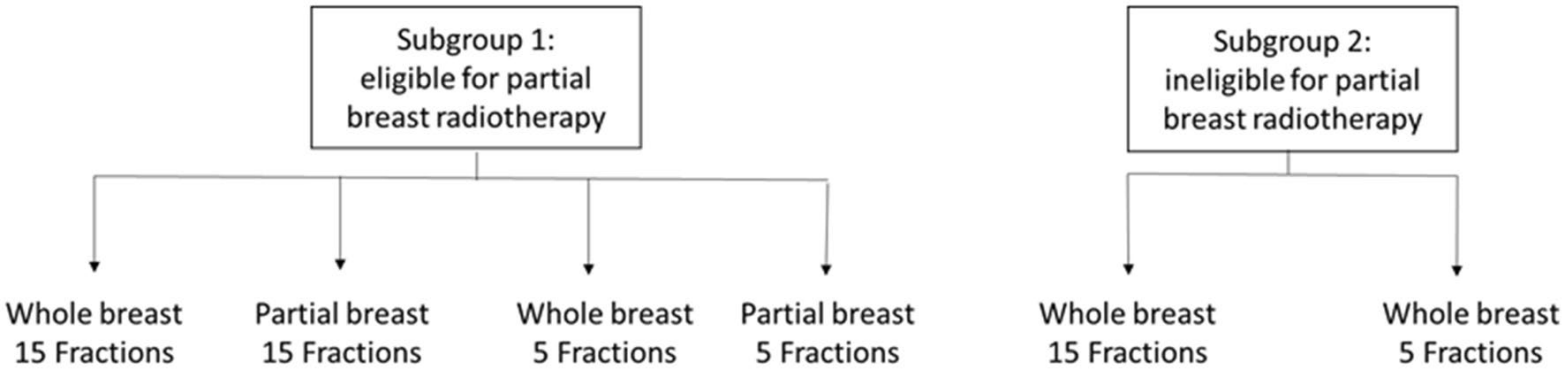
Decision tree for radiotherapy treatment period

**Fig. 2:**
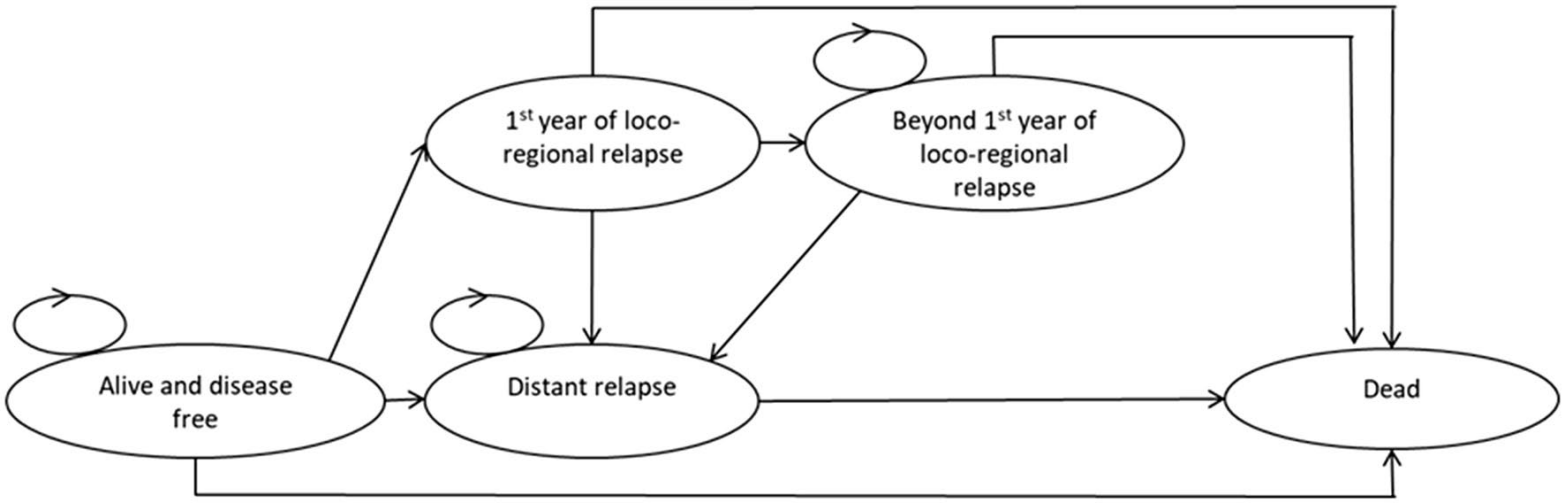
Markov model for post treatment outcomes

The structure of the economic model is illustrated in Fig. 1 and Fig. 2. The treatment period was modelled using a decision tree to estimate how the available radiotherapy modalities resulted in different treatment costs and side effects (Fig. 1). After the treatment period, a Markov model was used to estimate the long-term costs and health consequences (Fig. 2).

The analysis followed the reference case outlined by NICE in which direct health effects and costs which fall on the NHS were considered [14], and a discount rate of 3.5% per annum was used for both costs and health effects. Costs were expressed for a base year of 2019, adjusting for health care inflation [15]. Health impacts were captured using quality-adjusted life years (QALYs).

Model validation was carried out using the TECH-VER checklist [16]. The model was developed and run using R Statistical programming language and was based on the R code from the DARTH modelling group [17, 18].

A hypothetical cohort of patients entered the model following local tumour excision, and received one of the possible radiotherapy treatments, which differed in terms of resource use and likelihood of inducing acute skin reactions. Following radiotherapy, patients began in the “alive and disease free” health state and over time were at risk of locoregional relapse, distant relapse, or death. The arrows represent the possible transitions between states, and circular arrows indicate the possibility of staying in the same state for multiple cycles.

Locoregional relapse was modelled instead of local relapse (the primary outcome in FF and IL) in order to capture a wider range of outcomes in the economic model. Patient subgroup and radiotherapy modality determine how patients move through states over time. The Markov model cycle length was one year with half-cycle correction.

### Populating the model

Time to locoregional relapse, distant relapse, and some cost and health outcome model parameters were estimated from FF and IL individual patient data. All individual patient data analysis was carried out using Stata 16 [19]. Remaining model parameters were informed by the wider literature as detailed in Table 2 and Table 3.

**Table 2:** Calculation of radiotherapy costs

Input	Value	Source
Radiotherapy delivery		
Radiotherapy planning cost	£315	[21]
Cost of delivering one fraction of RT	£124	[21]
Percentage receiving breath hold with whole-breast radiotherapy	25%	Expert opinion
Percentage receiving breath hold with partial breast radiotherapy	10%	Expert opinion
Increase in fraction costs associated with breath hold	30%	Expert opinion, [22, 23]

Treatment	Formula: (unit cost) x (units per patient) x (proportion of patients)	Cost
WB15F		
Planning cost		£315.00
RT without breath hold	£124 × 15 × 75%	£1,395.00
RT with breath hold	(£124 × 1.3) × 15 × 25%	+ £604.50
Total		£2,314.50
WB5F		
Planning cost		£315.00
RT without breath hold	£124 × 5 × 75%	£465.00
RT with breath hold	(£124 × 1.3) × 5 × 25%	+ £201.50
Total		£981.50
PB15F		
Planning cost		£315.00
RT without breath hold	£124 × 15 × 90%	£1,674.00
RT with breath hold	(£124 × 1.3) × 15 × 10%	+ £241.80
Total		£2,230.80
PB5F		
Planning cost		£315.00
RT without breath hold	£124 × 5 × 90%	£558.00
RT with breath hold	(£124 × 1.3) × 5 × 10%	+ £80.60
Total		£953.60

*RT* Radiotherapy, *WB15F* Whole breast 15 fractions, *WB5F* Whole breast 5 fractions, *PB15F* Partial breast 15 fractions, *PB5F* Partial breast 5 fractions

**Table 3:** Input values for use in decision model

Input	Mean	Standard error	Distribution	Source
Population characteristics				
Average age at beginning of model				
Subgroup 1	63 years	–	–	IL
Subgroup 2	60 years	–	–	FF
Acute side effects during treatment				
Costs of treating adverse skin reactions for states: RTOG0, RTOG1, RTOG2a, RTOG2b, RTOG3	£0, £0, £132, £132, £136	–	–	Expert opinion, [44]
Probability of RTOG states: RTOG0, RTOG1, RTOG2a, RTOG2b, RTOG3				
15 Fraction radiotherapy	0%, 32%, 27.5%, 27.5%, 14%	–	Dirichlet	[20]
5 Fraction radiotherapy	6%, 62%, 13.5%, 13.5%, 6%	–	Dirichlet	[20]
Transition rate between states				
Alive disease free to locoregional relapse: 15F				
Subgroup 1	0.0022	0.0008	Exponential	IL
Subgroup 2	0.0077	0.0013	Exponential	FF
Alive disease free to distant relapse: 15F				
Subgroup 1	0.0032	0.0009	Exponential	IL
Subgroup 2	0.0132	0.0018	Exponential	FF
Alive and disease free to death	Variable -dependant on age	–	–	[25]
Locoregional relapse to distant relapse	0.0515	0.0045	Exponential	[28]
Locoregional relapse to death	Variable—dependant on age	–	–	[25]
Distant relapse to death			Exponential	[26]
Subgroup 1	0.2196	0.5102		
Subgroup 2	0.21	0.5102		
Relative treatment effect estimates				
Alive disease free to locoregional relapse: 5F vs 15F	0.66	0.167	Log normal	[6]
Alive disease free to locoregional relapse: PB vs WB	0.88	0.510	Log normal	[7]
Health care costs				
Alive and disease free (annual)				
Subgroup 1	£1,216	£82	Gamma	FF
Subgroup 2	£1,412	£68	Gamma	FF
Additional costs of 1st year alive and disease free	£402	£64	Gamma	FF
1st year of locoregional relapse				
Treatment costs	£4,241	± 20%	Gamma	[35–37, 40]
Supportive care costs	£2,995	± 20%	Gamma	[38, 40]
Beyond 1st year locoregional relapse (annual)	£2,139	± 20%	Gamma	[38, 40]
Distant relapse (annual)	£13,426	± 20%	Gamma	[39, 40]
Health-related quality of life				
Alive and disease free				
Subgroup 1	0.8302	0.0040	Gamma	IL
Decrement for subgroup 2	0.0150	0.0100	Gamma	FF
Locoregional relapse				
Subgroup 1	0.8302	0.0040	Gamma	IL
Decrement for subgroup 2	0.0150	0.0100	Gamma	FF
Decrement for distant relapse relative to alive and disease free	0.3030	0.1550	Gamma	[29]
Quality of life decrement with age	Variable -dependant on age	–	–	[43]

*RTOG* Radiation Therapy Oncology Group, *15F* 15 Fractions, *5F* 5 fractions, *WB* whole breast, *PB* partial breast

### Radiotherapy treatment period

Costs of delivering radiotherapy and costs of managing acute side effects were considered. Patients were assumed to receive treatment as specified in the trial protocols of FF and IL [6, 7]. Radiotherapy costs were applied at model entry and are calculated as shown in Table 2. As radiotherapy delivery costs were not recorded in the trials, resource use was informed by expert opinion. The main difference in cost between partial and whole-breast radiotherapy was assumed to result from reduced use of cardiac breath hold with PB. This procedure aids targeting of radiotherapy and reduces the radiation dose to cardiac tissue when the tumour is in the vicinity of the heart. Expert opinion was used to inform the proportions receiving cardiac breath hold (see Table 2).

Quality of life was assumed to be the same across all treatments during the treatment period due to an absence of preference-based quality of life data for this period. Costs relating to treating acute adverse skin reactions during treatment were included (see Table 3). It was assumed that these are one-off costs which depend on the severity of adverse effects as measured by worst Radiation Therapy Oncology Group (RTOG) score observed during treatment [20].

### Post radiotherapy period

#### Transition probabilities

The baseline rate of transition from the alive and disease-free state to locoregional and distant relapse was estimated from FF and IL. For subgroup 1, we estimated the rates of relapse with WB15F using observations from IL (*n =* 674). For subgroup 2, the rates of relapse with WB15F were estimated by identifying a PB ineligible subgroup in FF (*n =* 753). For the primary analysis, exponential parametric survival models were chosen for both locoregional and distant relapse as it fits the data well. This model assumed a constant rate of relapse over time [24].

It was assumed that patients who have not experienced a distant relapse are at the same risk of all-cause mortality as the age-matched general population [25]. Patients who had a distant relapse were at increased risk of death. Risks were based on a large French study of metastatic breast cancer. For the base case, risk of death was adjusted for average subgroup age and based on the hormone receptor + and HER2− molecular subtype as this was the most common in FF/IL, and the impact of using alternative subtypes was investigated in sensitivity analyses [26]. It was assumed that increased mortality from breast cancer occurs by passing through distant relapse. The UK rate of breast cancer mortality was removed from all-cause mortality to avoid double counting [27].

The rate of transition from locoregional to distant relapse was from a Dutch breast cancer study [28]. It was assumed that radiotherapy modality did not affect the rate from locoregional to distant relapse or the mortality risk post distant relapse.

#### Treatment effects

To model the rates of locoregional recurrence for PB15F and WB5F, we applied the hazard ratio for locoregional recurrence reported in FF and IL to the subgroup 1 baseline rate of locoregional recurrence. To estimate the relapse rates in the unobserved treatment option (PB5F), treatment effects were assumed to combine without synergy or attenuation (i.e. additivity on the log scale) [29–31]. For subgroup 2, we applied the locoregional hazard ratio from FF to estimate locoregional recurrence rates for WB5F [2].

We assumed a common pattern of transition from alive and disease free to distant recurrence across arms. This assumption was based on the clinical argument that radiotherapy is a local treatment and so its causal impact on distant recurrence (if any) should only occur through reducing local–regional recurrences. We explored the impact of this assumption in a sensitivity analysis.

Treatment effects were assumed to persist over time. For all survival outcomes, the correlations between baseline event rates and relative effects were maintained.

#### Costs

Only the FF questionnaire was used to estimate costs as it was considered more complete than the IL cost questionnaire. It covered activities related and unrelated to breast cancer such as general practitioner costs, nursing costs, and hospitalisations. Unit costs were applied to resource use to construct per patient costs [15, 21, 32–34].

Costs for the alive and disease-free state were estimated from FF using a generalised estimating equations (GEE) panel model [19]. 1,179 patients were included in the FF economic sub-study and followed over time resulting in *n =* 4,519 observations. A dummy variable was included to capture differences in costs between subgroups and elevated costs in the first 6 months after treatment. Costs were found not to differ by treatment option or age.

Costs for the remaining health states were sourced from the wider literature as there were insufficient observations to estimate them from FF. The first year of locoregional relapse was associated with one-off mastectomy costs in addition to supportive care costs [35–38]. Following the first year of locoregional relapse, supportive care is assumed to consist of one GP visit and one mammogram per year [38]. Supportive care and treatment costs for distant relapse were sourced from a UK study of 77 women [39].

Costs unrelated to breast cancer were added to the breast cancer costs for locoregional and distant relapse health states to make them consistent with the inclusion of related and unrelated costs in the FF resource use questionnaire [40].

### Health-related quality of life (HRQoL)

HRQoL was estimated for the alive and disease-free state using data from both FF and IL. It was found that HRQoL did not statistically differ by treatment option, age or change systematically over time but did differ by subgroup. HRQoL was estimated for subgroup 1 using data from IL (*n =* 653) with the difference in HRQoL between subgroups estimated from FF (*n =* 1,044). HRQoL was measured using the EQ-5D-5L questionnaire in FF and EQ-5D-3L in IL. For consistency, the 5L questionnaire was mapped to 3L index scores [41, 42]. A generalised linear model (GLM) was used to model disutility based on the first wave of data after treatment in each study (3 months for FF and 6 months for IL) [19].

It was assumed that HRQoL post locoregional relapse was the same as for the alive and disease-free state. The decrement in HRQoL with distant relapse was taken from a previous radiotherapy model [29]. The decline in HRQoL with age was based on a Health Survey for England study [43].

### Cost-effectiveness

The model followed a cohort of patients aged 63 (subgroup 1) or 60 (subgroup 2) for 50 years after finishing radiotherapy. Over this period, discounted costs and QALYs were calculated for each treatment option. To estimate incremental cost-effectiveness ratios (ICERs), dominated treatment options were identified and excluded from further consideration. These were options which had lower expected QALYs and higher expected costs than a comparator. To compare the remaining (non-dominated) options, we ordered treatments from cheapest to most expensive, dividing the additional expected costs by the additional expected QALYs.

ICERs were compared to a cost-effectiveness threshold of £15,000/QALY. This is the value used by the Department of Health in the UK to represent the marginal rate at which NHS activities generate QALYs [45, 46]. The option which had the highest estimated QALY gain and an ICER below £15,000/QALY was considered the cost-effective option.

### Sensitivity analysis

Uncertainties in model inputs due to limited sample size were reflected in distributions for each input (see Table 3). The joint impact of this uncertainty on costs and health consequences was explored through probabilistic sensitivity analysis (PSA). PSA involved (i) drawing input values according to their relative plausibility (ii) entering these input values into the model to calculate costs and health outcomes (iii) storing results and (iv) repeating steps i-iii 10,000 times.

One-way sensitivity analysis was used to explore the sensitivity of results to one-at-a-time changes in individual inputs and assumptions (see Table 4).

**Table 4:** Results for base case and sensitivity analyses in subgroups 1 and 2. All analyses are based on 10,000 probabilistic simulations

Scenario	Subgroup 1 (eligible for partial breast radiotherapy)				Subgroup 2 (not eligible for partial breast radiotherapy)	
	Whole breast 15 Fractions	Partial breast 15 Fractions	Whole breast 5 Fractions	Partial breast 5 Fractions	Whole breast 15 Fractions	Whole breast 5 Fractions
	ICER (probability treatment ICER < £15,000/QALY)					
0. Base case	Dom (0%)	Dom (0%)	Dom (38%)	£0 (62%)	Dom (0%)	£0 (100%)
1. Distant recurrence hazard ratio reported in FF and IL	Dom (14%)	£15,050 (24%)	Dom (25%)	£0 (37%)	£3,937 (76%)	£0 (23%)
2. All treatment effects maintained for 10 years	Dom (0%)	Dom (0%)	Dom (37%)	£0 (63%)	Dom (0%)	£0 (100%)
3. All treatment effects maintained for 5 years	Dom (0%)	Dom (0%)	Dom (35%)	£0 (65%)	Dom (0%)	£0 (100%)
4. Mortality rate following distant recurrence based on HER2 + population [26]	Dom (0%)	Dom (0%)	Dom (38%)	£0 (62%)	Dom (0%)	£0 (100%)
5. Mortality rate following distant recurrence based on TNBC (HR- & HER2-) population [26]	Dom (0%)	Dom (0%)	Dom (38%)	£0 (62%)	Dom (0%)	£0 (100%)
6. Distant relapse costs reduced to £8,934 per year [47]	Dom (0%)	Dom (0%)	Dom (38%)	£0 (62%)	Dom (0%)	£0 (100%)
7. Distant relapse costs increased to £16,111 per year (20% increase)	Dom (0%)	Dom (0%)	Dom (38%)	£0 (62%)	Dom (0%)	£0 (100%)
8. Disutility resulting from distant relapse reduced to 0.26 [48]	Dom (0%)	Dom (0%)	Dom (38%)	£0 (62%)	Dom (0%)	£0 (100%)
9. Disutility resulting from distant relapse increased to 0.3636 (20% increase)	Dom (0%)	Dom (0%)	Dom (38%)	£0 (62%)	Dom (0%)	£0 (100%)
10. Use of breath hold assumed to not increase cost to deliver one fraction of radiotherapy (£124)	Dom (0%)	Dom (0%)	Dom (40%)	£0 (60%)	Dom (0%)	£0 (100%)
11. Use of breath hold assumed to double the cost to deliver one fraction of radiotherapy (£248)	Dom (0%)	Dom (0%)	Dom (33%)	£0 (67%)	Dom (0%)	£0 (100%)
12. Rate of adverse skin reactions set equal across all treatment options	Dom (0%)	Dom (0%)	Dom (38%)	£0 (62%)	Dom (0%)	£0 (100%)
13. Health-related quality of life weight during radiotherapy set to zero	Dom (0%)	Dom (0%)	Dom (38%)	£0 (62%)	Dom (0%)	£0 (100%)
14. Log normal survival model in which each participant only contributes their 1st event^a^	Dom (2%)	Dom (2%)	Dom (46%)	£0 (51%)	Dom (17%)	£0 (83%)

^a^Some individuals were recorded as having a distant relapse and locoregional relapse at the same time point. This scenario log-normal survival models were used to estimate time to first event, censoring individuals that experience more than one type of relapse

*CE* Cost-effective, *Dom* dominated, *RT* radiotherapy

## Results

**Fig. 3:**
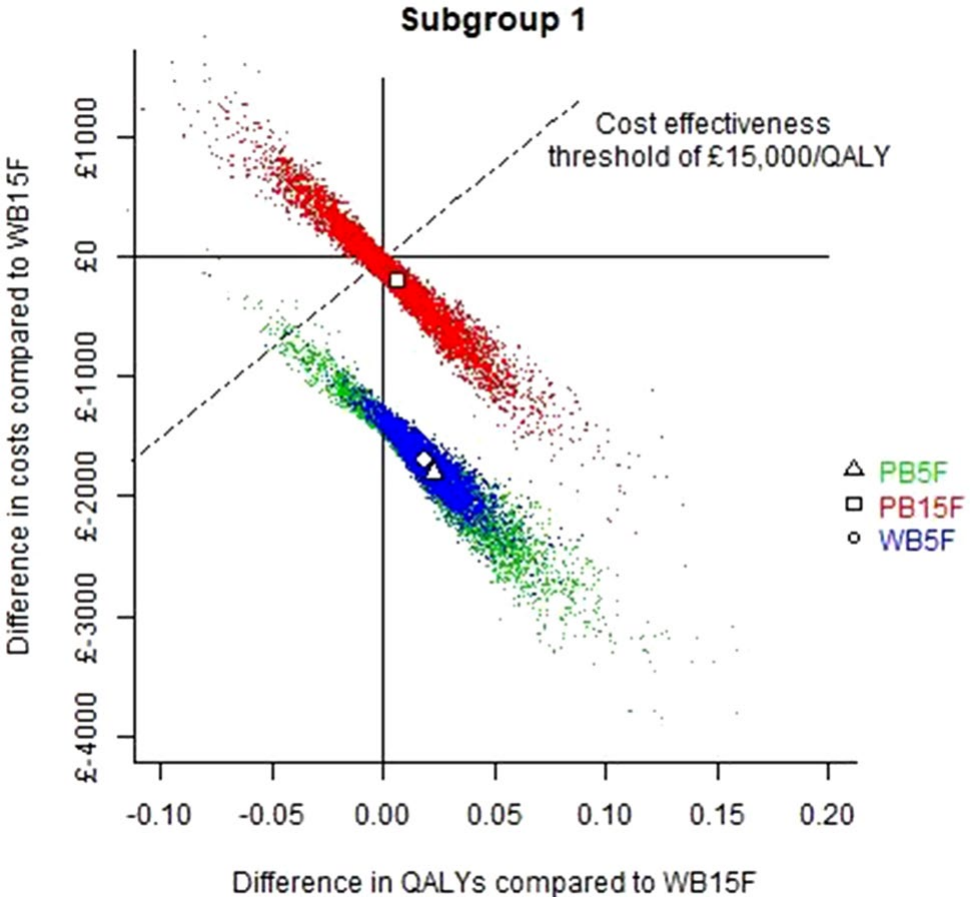
Base case cost-effectiveness plane for subgroup 1. All treatment options are relative to WB15F. WB15F = whole breast 15 fractions; WB5F = whole breast 5 fractions; PB15F = partial breast 15 fractions; PB5F = partial breast 5 fractions; QALYs = quality-adjusted life years

**Fig. 4:**
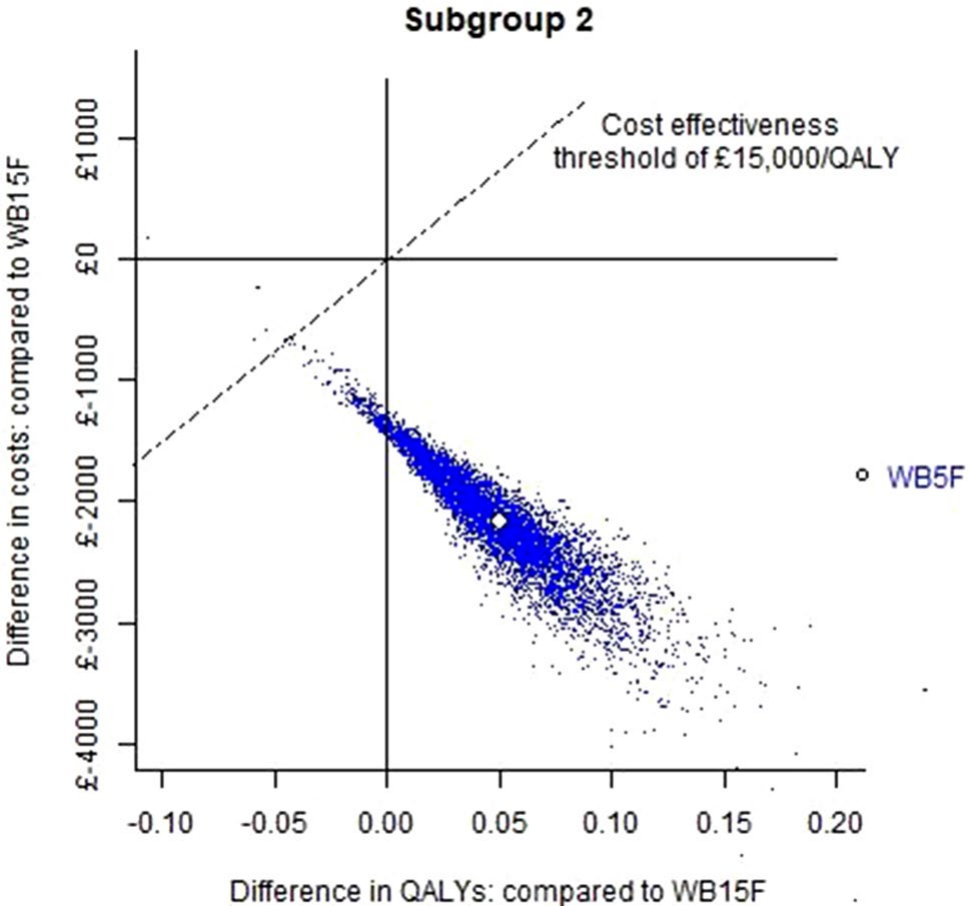
Base case cost-effectiveness plane for subgroup 2. WB15F is the reference treatment. WB15F = whole breast 15 fractions; WB5F = whole breast 5 fractions; QALYs = quality-adjusted life years

The expected costs and QALYs for each treatment in subgroup 1 are reported in Table 4. The probability of being the cost-effective option (at £15,000/QALY) was calculated for all 4 treatment alternatives in subgroup 1 (see Table 4). The probabilistic results are shown in the incremental cost-effectiveness plane in Fig. 3 (subgroup 1) and Fig. 4 (subgroup 2).

### Subgroup 1

Considering expected costs and QALYs, all treatment options were “dominated” by PB5F meaning PB5F was expected to have lower costs and greater QALYs than any of the alternatives. Across PSA simulations, there was a 62% chance that PB5F either dominated all alternatives or had an ICER below £15,000/QALY.

### Subgroup 2

WB5F dominated WB15F with expected cost savings of £2,162 (95% interval £1,282 to £3,169) and higher expected QALYs: 0.05 (95% interval 0.01 to 0.12). Across simulations, there was a 100% chance that WB5F either dominated WB15F or had an ICER below £15,000.

### Sensitivity analyses

Based on expected outcomes in subgroup 1, PB5F dominated all other options except when using the distant recurrence hazard ratio results reported in the trials. In this one scenario, PB15F compared with PB5F was expected to be more expensive by £1,014 (95% interval £-263 to £1,922) but also more effective by 0.07 additional QALYs (95% interval − 0.05 to 0.24). With a threshold of £15,000/QALY, PB5F was expected to have approximately the same cost-effectives as PB15F, but there remained a higher probability that PB5F was cost-effective compared to PB15F (56%).

For subgroup 2, WB5F dominated WB15F across all scenarios except when using the distant recurrence hazard ratio results reported in the trials. In this scenario, WB15F was expected to be more expensive at £472 (95% interval £-2214 to £2,942) more costly and more effective by 0.25 additional QALYs (95% interval -0.18 to 0.69). In this scenario, the expected ICER for WB15F was £1,899/QALY (probability ICER < £15,000 76%).

## Discussion

Across a range of scenarios, PB and 5F radiotherapy were expected to be cost-effective at a threshold of £15,000/QALY across both subgroups.

In subgroup 1 (patients eligible for partial breast radiotherapy), PB5F was expected to provide more QALYs and have lower costs compared to the other three alternatives. The expected cost savings were primarily due to reducing the number of fractions of radiotherapy. The improvement in QALYs was driven by the modest expected reduction in locoregional recurrences. This effect identified in IMPORT LOW was not statistically significant (hazard ratio 0.88, 95% interval 0.34–2.27) but had an influence on expected outcomes. Figure 3 illustrates how PB5F is associated with lower costs and greater QALYs. This figure also illustrates that the outcomes were broadly similar with the 5F therapies (WB5F and PB5F), indicating that gains were primarily from switching from 15 to 5F.

For subgroup 2 (patients not eligible for partial breast radiotherapy), WB5F was expected to provide more QALYs and have lower costs compared to WB15F. Again, cost savings were primarily due to reducing the number of fractions of radiotherapy. Figure 4 illustrates that the spread of points for WB5F lies completely to the south east of the £15,000 cost-effectiveness threshold, this indicates that the cost-effectiveness of WB5F was not associated with any parametric uncertainty.

Resource savings from reduced fractionation may enable the same number of patients to be treated with lower linear accelerator capacity therefore freeing capacity to treat breast and other cancers. Further, the benefits to patients would immediately be realised in terms of reduced burden of treatment, and this may have added benefits such as reduced exposure to COVID-19 as a result of attending hospital for treatment [49].

The one-way sensitivity analyses reported in Table 4 illustrated that these overall conclusions were robust to alternative inputs and assumptions. Across all scenarios, the 5F regimens remained the least costly alternative. Results were not sensitive to the molecular subtype population chosen to model mortality following distant recurrence [26]. This was because in this scenario *relative* rates of distant recurrence were assumed common across arms. In the scenario in which relative rates of distant recurrence were calculated using the hazard ratios reported in FF and IL, 5F was associated with fewer QALYs. With a cost-effectiveness threshold of £15,000/QALY, PB5F and PB15F were expected to be similarly cost-effective in subgroup 1, and in subgroup 2, WB5F was not expected to be the cost-effective alternative. The relatively lower QALYs associated with 5F in these scenarios were due to the HR for distant recurrence for 5F vs 15F estimated in FF. This estimate indicated a slightly higher rate of distant recurrence with 5F relative to 15F, HR = 1.27 (0.90 to 1.79). Though this was not statistically significant (*p =* 0.17), distant recurrence had a large impact on both morbidity and mortality and so drove differences in expected QALYs between treatment options.

As discussed in the methods section, the key assumption in the base case analysis is that there was a common pattern of transition from alive and disease free to distant recurrence across treatment options. This assumption was based on the clinical argument that radiotherapy is a local treatment with local effects. One alternative approach to estimate treatment effects would be to estimate the rate of any recurrence (distant or local or regional). This approach could be used to estimate baseline and relative effects across subgroups and radiotherapy modalities. This alternative approach was not carried out here in order to base the economic analysis on the published clinical results. Further, estimating the difference in any recurrence between arms would imply a common treatment effect on local, distant and regional recurrences which may not be clinically plausible. Even if this approach was taken, it would still be necessary to separate out the different outcomes as they have different costs and health consequences. A fully comprehensive approach may require a multi-state modelling framework and thus a different approach to both data collection and modelling [50, 51]. This approach would need to take account of recognised issues with effect identification [52, 53].

There were other limitations to analysis. The impact of a reduction in exposure of internal organs to harmful radiation with PB was not modelled in this study [7]. The impact of acute skin reactions was only captured by increased costs associated with treatment, the HRQoL impact was not captured due to lack of EQ-5D data during the treatment period. Including HRQoL impacts into the analysis would likely improve the relative benefits of reduced fractionation as 5F was observed to have fewer severe acute adverse reactions than 15F in Brunt et al., (2016) [20]. It is unlikely that longer term data would change this conclusion as absolute effects are very low, so would require dramatic shifts to change conclusions. Further, evidence form START and FAST indicate effects which are relatively stable at 5 and 10 years follow-up [54, 55]. The base case assumed known proportion of patients received cardiac breath hold (see Table 2); however, these rates will vary across settings. The impact of this explored in sensitivity analysis 10 and was found to have no impact on conclusions.

To our knowledge, no other studies have examined the cost-effectiveness of PB radiotherapy or 5F hypofractionation in a UK context. For the US system, two studies (Shah et al., 2013 and Sher et al., 2009) found that PB was cost-effective relative to whole-breast radiotherapy, this is in line with our conclusions [56, 57]. Deshmukh et al., (2017) compared whole-breast radiotherapy delivered over 5–7 weeks to radiotherapy delivered over 3–4 weeks and found that reducing fractionation (and therefore duration of radiotherapy) dominated higher fractionation [58]. This is in line with our base case results. Though favourable to PB, the results of this analysis do not pertain directly to more complex intensity-modulated radiation therapy (IMRT) such as that utilised in the Florence protocol [59]. A full assessment of this approach would require bespoke costing and, most importantly, an assessment of efficacy and safety for this technology. The aim of IMRT is to significantly reduce the partial breast volume. It is unclear whether the same very low levels of ipsilateral breast tumour relapse observed with PB in IMORT LOW could be achieved with smaller volumes.

Based on the results presented, hypofractionation to 5F and PB radiotherapy modalities offer potentially significant benefits to the UK health system. This analysis supports efforts to widen adoption of these innovative modalities.

## Supplementary Information

Below is the link to the electronic supplementary material.

Supplementary file1 (DOC 304 kb)

## Data Availability

Enquiries about data availability should be directed to the authors.
