# Supplementary material for: Cost-effectiveness of 5 fraction and partial breast radiotherapy for early breast cancer in the UK: model-based multi-trial analysis
Source: Breast Cancer Res Treat. 2022 Nov 17;197(2):405–16. doi: 10.1007/s10549-022-06802-1 (PMC9672618; doi:10.1007/s10549-022-06802-1)
Supplement: Supplementary file 1 — Supplementary file1 (DOC 304 kb) [file 10549_2022_6802_MOESM1_ESM.doc]

# Cost-effectiveness of hypofractionation and partial breast radiotherapy for early breast cancer in the UK: Supplementary material

Contents

[Statistical analysis 2](#__RefHeading___Toc63697000)

[Average age of the cohort at the start of the model 2](#__RefHeading___Toc63697001)

[Estimate QoL for health states 3](#__RefHeading___Toc63697002)

[Calculate EQ5D index values 4](#__RefHeading___Toc63697003)

[Estimating quality of life model 6](#__RefHeading___Toc63697004)

[Results for use in economic model 7](#__RefHeading___Toc63697005)

[Survival analysis 8](#__RefHeading___Toc63697006)

[Models to estimate 8](#__RefHeading___Toc63697007)

[Parametric survival modelling 8](#__RefHeading___Toc63697008)

[Loco-regional recurrence 9](#__RefHeading___Toc63697009)

[Distant recurrence 12](#__RefHeading___Toc63697010)

[Estimate costs of health states 16](#__RefHeading___Toc63697011)

[Summary of trial data collected on resource use 16](#__RefHeading___Toc63697012)

[Attach cost data to resource use 17](#__RefHeading___Toc63697013)

[Classification of resource use observations into relevant health states 18](#__RefHeading___Toc63697014)

[Estimating models 20](#__RefHeading___Toc63697015)

[Model inputs 22](#__RefHeading___Toc63697016)

[Inputs estimated from FAST forward / IMPORT LOW 22](#__RefHeading___Toc63697017)

[Treatment costs 23](#__RefHeading___Toc63697018)

[During treatment 24](#__RefHeading___Toc63697019)

[After treatment 25](#__RefHeading___Toc63697020)

[Estimating costs unrelated to breast cancer 29](#__RefHeading___Toc63697021)

[Model assumptions 31](#__RefHeading___Toc63697022)

[Model validation 33](#__RefHeading___Toc63697023)

[Validation of model predictions against trial data 33](#__RefHeading___Toc63697024)

[Model 1 33](#__RefHeading___Toc63697025)

[Model 4 34](#__RefHeading___Toc63697026)

[TECH VER checklist (Details reported in Büyükkaramikli et al, 2019) 36](#__RefHeading___Toc63697027)

[References 42](#__RefHeading___Toc63697028)

# Statistical analysis

This section describes the statistical analysis carried out to inform the economic model. It is composed of 4 sections:

- Calculating average age at the start of the model
- Estimating quality of life (QoL) for health states
- Estimating survival models
- Estimating the costs of health states

## Average age of the cohort at the start of the model

Subgroup 1: IMPORT LOW the average age, pooling all treatment arms (n = 2,016) was 63. Subgroup 2: FAST Forward the average age of those ineligible for partial breast radiotherapy, pooling all treatment arms (n = 2,239) was 60.

## Estimate QoL for health states

Quality of life (QoL) was captured in each study using two versions of the EuroQol instrument. The EQ-5D 5 level questionnaire was used in FAST Forward, and the EQ-5D 3 level questionnaire was used in IMPORT LOW.

In FAST Forward EQ-5D-5L data was collected in the PROMS sub-study (n=1,179) at baseline (before randomisation or radiotherapy), 3, 6 months post treatment and 1,2 and 5 years post randomisation. In IMPORT LOW EQ-5D-3L data was collected on in a subset of patients (n = 1,290). Data was collected in a subset of participating centres. Data collected at baseline (prior to randomisation), 6 months, and 1, 2, and 5 years post randomisation.

It is necessary to classify each time point for each patient into a mutually exclusive set of health states. For initial investigation, we classify into: alive and disease free, any recurrence (loco-regional recurrence, distant recurrence, new primary tumour) and death. For example, a patient is defined as being alive and disease free at a particular time point if the date of the EQ-5D questionnaire is before the date of: loco-regional recurrence, distant recurrence, new primary tumour or death. If the patient did not experience any of these events during the follow up period of the trial, then the patient can be classified as alive and disease free at all time points.

For IMPORT LOW, the date of EQ-5D questionnaire delivery was not recorded. In Fast Forward 95% of the EQ-5D questionnaires were delivered within 2 months of the scheduled date. This information was used to define a calliper range around the scheduled date of the questionnaire for IMPORT LOW. For IMPORT LOW, if a patient progressed within +/- 2 months of the scheduled date of the EQ5D questionnaire, they were classified as progressed.

EQ-5D data is considered “missing” if there is 1) a missing questionnaire, 2) an incomplete questionnaire or 3) the questionnaire for a particular time point has been filled in outside the appropriate window. The appropriate window is defined as the time between the midpoint of the previous data collection date and midpoint of the next data collection date. E.g. 2 year data is missing if there has been no data entered between 1 year + 6 months and 3 years + 6 months. This approach follows Woods et al., 2018 [1]. Where the date of the EQ-5D questionnaire was missing, patients were classified into health states by comparing the time of first progression to an estimate of the questionnaire date.

### Calculate EQ5D index values

EQ-5D-5L was collected in FAST forward, EQ-5D-3L was collected in IMPORT LOW. To combine data these trials should converted to a common value set. NICE’s current advice is to map 5L scores to 3L using the method described by van Hout et al., (2012) [2]. This was done for FAST Forward which collected 5L. The eqd5 package by J. M. Ramos-Goñi and O. Rivero-Arias (2011) was used to calculate UK EQ5D3L index scores from the IMPORT LOW data [3].

**Step 1: define valid EQ5D observations**

**Step 2: classify patients into disease states**

**Step 3: calculate % missing EQ5D index scores by disease state**

For each disease state and time point we calculated the number (and %) of missing EQ5D index observations. This analysis shows the degree of missing data for each health state and informs which health states have sufficient data to estimate quality of life and what missing data methods (if any) are required.

#### FAST Forward

Table 1 below shows the number of observations and missing data patterns across health states in FAST Forward.

Table 1

|  | Health state | | | | | | | | | |  |  |  |
| --- | --- | --- | --- | --- | --- | --- | --- | --- | --- | --- | --- | --- | --- |
|  | Alive and disease free | | | |  | Any recurrence | | | |  | Death |  | Sum |
|  | Pat | Missing | | Mean |  | Pat | Missing | | Mean |  | Pat |  | Pat |
| Baseline | 1,178 | 32 | (2.7%) | 0.800 |  | 1 | 0 | (0.0%) | 0.877 |  | 0 |  | 1,179 |
| Month 3 | 1,157 | 113 | (9.8%) | 0.778 |  | 13 | 12 | (92.3%) | 0.736 |  | 9 |  | 1,179 |
| Month 6 | 1,163 | 111 | (9.5%) | 0.770 |  | 5 | 2 | (40.0%) | 0.791 |  | 11 |  | 1,179 |
| Year 1 | 1,158 | 108 | (9.3%) | 0.788 |  | 7 | 3 | (42.9%) | 0.657 |  | 14 |  | 1,179 |
| Year 2 | 1,129 | 155 | (13.7%) | 0.777 |  | 22 | 5 | (22.7%) | 0.755 |  | 28 |  | 1,179 |
| Year 5 | 1,069 | 258 | (24.1%) | 0.782 |  | 46 | 1 | (2.2%) | 0.742 |  | 64 |  | 1,179 |
| Pat=number of patients in health state (including missing), Missing=number and proportion of observations with missing EQ-5D index score in health state, Mean=average EQ-5D index score | | | | | | | | | | | | | |

Table 2 shows the summary statistics for alive and disease free health state in FAST Forward.

Table 2

| Variable | Alive and disease free | | | | |  |
| --- | --- | --- | --- | --- | --- | --- |
|  | Obs | Mean | Std Err | Min | Max |  |
| EQ-5D-3L utility score (cross-walked) |  |  |  |  |  |  |
| Baseline | 1,146 | 0.800 | 0.174 | -0.209 | 1 |  |
| Month 3 | 1,044 | 0.778 | 0.180 | -0.181 | 1 |  |
| Month 6 | 1,052 | 0.770 | 0.186 | -0.594 | 1 |  |
| Year 1 | 1,050 | 0.788 | 0.191 | -0.594 | 1 |  |
| Year 2 | 974 | 0.777 | 0.196 | -0.161 | 1 |  |
| Year 5 | 811 | 0.782 | 0.215 | -0.594 | 1 |  |
| Std Err=standard error. | | | | | | |

#### IMPORT LOW

The table below shows the number of observations and missing data patterns across health states in IMPORT LOW.

Table 3

|  | Health state | | | | | | | | | |  |  |  |
| --- | --- | --- | --- | --- | --- | --- | --- | --- | --- | --- | --- | --- | --- |
|  | Alive and disease free | | | |  | Any recurrence | | | |  | Death |  | Sum |
|  | Pat | Missing | | Mean |  | Pat | Missing | | Mean |  | Pat |  | Pat |
| Baseline | 847 | 43 | (5.1%) | 0.820 |  | 0 | 0 | - | - |  | 0 |  | 847 |
| Month 6 | 844 | 191 | (22.6%) | 0.831 |  | 1 | 1 | (100.0%) | - |  | 2 |  | 847 |
| Year 1 | 838 | 204 | (24.3%) | 0.838 |  | 4 | 2 | (50.0%) | 0.468 |  | 5 |  | 847 |
| Year 2 | 829 | 166 | (20.0%) | 0.827 |  | 9 | 1 | (11.1%) | 0.700 |  | 9 |  | 847 |
| Year 5 | 760 | 175 | (23.0%) | 0.827 |  | 35 | 10 | (28.6%) | 0.823 |  | 52 |  | 847 |
| Pat=number of patients in health state (including missing), Missing=number and proportion of observations with missing EQ-5D index score in health state, Mean=average EQ-5D index score | | | | | | | | | | | | | |

Descriptive statistics for the alive and disease-free state over time are reported in Table 4.

Table 4

| Variable | Alive and disease free | | | | |  |
| --- | --- | --- | --- | --- | --- | --- |
|  | Obs | Mean | Std Err | Min | Max |  |
| EQ-5D-3L utility score (cross-walked) |  |  |  |  |  |  |
| Baseline | 804 | 0.820 | 0.190 | -0.184 | 1 |  |
| Month 6 | 653 | 0.831 | 0.199 | -0.181 | 1 |  |
| Year 1 | 634 | 0.838 | 0.194 | -0.319 | 1 |  |
| Year 2 | 663 | 0.827 | 0.209 | -0.126 | 1 |  |
| Year 5 | 585 | 0.827 | 0.209 | -0.181 | 1 |  |
| Std Err=standard error. | | | | | | |

### Estimating quality of life model

For subgroup 1, a model was estimated based on all patients in IMPORT LOW (subgroup 1). FAST Forward data was used to estimate the average QoL differential between subgroup 1 and subgroup 2.

#### Choosing models for panel data analysis

A generalised liner model (GLM) based on the wave of data closest to treatment was used for simplicity as time was found to have no statistical impact on results. A gamma distribution for disutility was used as this fits the range utility data. An identity link was chosen.

A complete case analysis (CCA) was chosen as the degree of missing data in the study was low, less than 25% at any timepoint for both studies. This implies a missing completely at random assumption (MCAR) i.e. missingness is independent of both observed and unobserved values (Faria et al., 2014) [4].

As we want to inform the average utility for the alive and disease-free state, data was restricted to only those known to be in the alive and disease-free state (see previous section for more details).

#### FAST Forward

Using the full sample (6,143 observations), we estimated a GLM model. This model included a dummy for the alive and disease-free state, a dummy for eligibility to partial breast radiotherapy, a time dummy equal to one for all post-treatment time points, all possible interactions between the previous dummies and a dummy for the treatment arm. The marginal effect of being eligible for partial breast radiotherapy for the subgroup of patients who were in the alive and disease-free state after treatment was equal to -0.015 (SE=0.010, p-value=0.149). The average EQ-5D score for patients who were ineligible for was partial breast radiotherapy and in the alive and disease-free state after treatment was 0.227 (SE=0.006, p-value<0.001), while the same average for eligible patients was 0.212 (SE=0.008, p-value<0.001).

#### IMPORT LOW

A GLM with age as a covariate was fitted. The effect of age was significant at 5% level. A dummy for treatment group and a non-liner effect of age were included in the model but neither were statistically significant and so were not included in the model. The mean disutility at this age with standard error are 0.1698468 and 0.004045 respectively. This corresponds to a utility of 0.83.

### Results for use in economic model

##### Subgroup 1

The average disutility for the alive and disease-free state for subgroup 1 (eligible for partial breast radiotherapy) was estimated from IMPORT LOW with mean and standard error of 0.1698468 and 0.004045 respectively (note this value is for the average age in this group: 63).

##### Subgroup 2

The analysis of FAST Forward provided an estimate for the difference in QoL between subgroup 1 and subgroup 2. This has a mean and standard error of 0.015 and 0.010 respectively. (note this differential includes the differences in baseline age between the subgroups).

## Survival analysis

### Models to estimate

Loco-regional recurrence and distant recurrence outcomes are estimated using separate models. This is the method used in the clinical analyses in which separate Cox models are fit to loco-regional and distant recurrence.

### Parametric survival modelling

To choose an appropriate statistical model, the algorithm outlined in DSU guidance (2013) was used [5]. It was found that exponential and log-normal survival models fit the data best. This resulted in four survival models which were estimated for both loco-regional recurrence and distant recurrence (i.e. 8 models in total).

Model 1: Treatment effect estimates taken from clinical papers, baseline event rates from control arms

This case uses exponential survival models fit to control arms for both loco-regional recurrence and distant recurrence. These survival outcomes are estimated on the raw data observed in FAST Forward and IMPORT LOW. The hazard ratios for each outcome are taken directly from the clinical papers.

Model 2: Re-estimation of treatment effects as part of a full parametric model

This is the same as model 1 but the hazard ratios are re-estimated simultaneously with the baseline model. Therefore, they differ slightly from those reported in the clinical trial. An exponential model is again utilised to model baseline event rates. The benefit of this approach is that the baseline model and relative effect model are consistent.

Model 3: Adjust estimates of time to recurrence for double counting

In the base case loco-regional recurrence and distant recurrence are treated completely separately, as in the clinical trial. However, this will overestimate the rate of recurrences generally as some individuals are recorded as having a distant recurrence and loco-regional recurrence at the same time point. In the base case analysis these are counted as both a distant and loco-regional recurrence. This is due to an inconsistency between the decision model and the statistical model used to analyse the data. In this scenario we use base case inputs and use exponential survival models but adjust HR for double counting. Both baseline risks and hazards ratios are estimated in one consistent parametric model.

Model 4: Estimate survival models using log normal model (and adjust for double counting)

The exponential survival models used in the base case and in all of the above sensitivity analyses assume a constant hazard. This means that the risk of loco-regional (or distant) recurrence is constant over time i.e. the risk of recurrence is the same one year after treatment as 20 years after treatment. This assumption may not be reasonable. Another survival which fit the observed data well was the log-normal model, this allows for the risk of recurrence to increase then decrease over time. As in the sensitivity analysis above, outcomes were adjusted for double counting to improve consistency between the decision model and survival model.

### Loco-regional recurrence

#### Summary of point estimates

The table below summarises the point estimates for the treatment effects in each of the four models.

Table 5

| Local-regional recurrence: eligible to partial breast radiotherapy | | | | | | | | |
| --- | --- | --- | --- | --- | --- | --- | --- | --- |
| Model | |  | Relative treatment effect: 5F vs 15F (source: FF) | |  | Relative treatment effect: partial vs whole (source: IL) | |  |
|  |  |  | Hazard ratio | Acceleration factor |  | Hazard ratio | Acceleration factor |  |
|  |  |  | Coeff | Coeff |  | Coeff | Coeff |  |
| 1 | Exponential model, fit to control arms + HR from clinical trial |  | 0.66 | - |  | 0.88 | - |  |
| 2 | Exponential model, simultaneous estimation of baseline + HR |  | 0.68 | 0.39 |  | 0.89 | 0.12 |  |
| 3 | Exponential model, simultaneous estimation of baseline + HR with adjustment for double counting |  | 0.54 | 0.61 |  | 0.89 | 0.12 |  |
| 4 | Lognormal model, simultaneous estimation of baseline + AF with adjustment for double counting |  | - | 0.48 |  | - | 0.08 |  |

Note that Table 5 reports the acceleration factor for models 2 to 4. This is another way of expressing differences between treatments. It is reported because model 4 requires this expression of relative effects. Reporting this allows comparison of results across models.

#### Parameters

**IMPORT LOW: Estimation of baseline only, no adjustment for double counting**

The parameters below correspond to the subgroup 1 baseline estimates for model 1

Log scale - Model fitted on control group (WB15F) only (n = 674)

Table 6

| Model | Constant | |  |
| --- | --- | --- | --- |
|  | Coeff | SE |  |
| Exponential | -6.0686 | 0.3333 |  |

**FAST Forward: Estimation of baseline only, no adjustment for double counting**

The parameters below correspond to the subgroup 2 baseline estimates for model 1

Log scale - Model fitted on partial breast eligible section of control group (WB15F) only (n = 753)

Table 7

| Model | Constant | |  |
| --- | --- | --- | --- |
|  | Coeff | SE |  |
| Exponential | -4.8752 | 0.1826 |  |

**IMPORT LOW: Simultaneous estimation of baseline and treatment effect, with no adjustment for double counting**

The parameters below correspond to model 2

Log scale - Model fitted on full sample (n = 1343).

Table 8

| Model | Constant | |  | Treatment | |  |
| --- | --- | --- | --- | --- | --- | --- |
|  | Coeff | SE |  | Coeff | SE |  |
| Exponential | -6.0686 | 0.3333 |  | -0.1220 | 0.4859 |  |

Table 9

| Exponential variance-covariance matrix | | |  |
| --- | --- | --- | --- |
|  | Treatment | Constant |  |
| Treatment | 0.2361 |  |  |
| Constant | -0.111111 | 0.111111 |  |

**FAST Forward: Simultaneous estimation of baseline and treatment effect, with no adjustment for double counting**

The parameters below correspond to model 2

Log scale - Model fitted on full sample (n = 2723).

Table 10

| Model | Constant | |  | Subgroup | |  | Treatment | |  |
| --- | --- | --- | --- | --- | --- | --- | --- | --- | --- |
|  | Coeff | SE |  | Coeff | SE |  | Coeff | SE |  |
| Exponential | -4.9097 | 0.1736 |  | -0.6557 | 0.2566 |  | -0.3878 | 0.2414 |  |

Table 11

| Exponential variance-covariance matrix | | | |  |
| --- | --- | --- | --- | --- |
|  | Treatment | Subgroup | Constant |  |
| Treatment | 0.0582923 |  |  |  |
| Subgroup | 0.00001 | 0.065863 |  |  |
| Constant | -0.023813 | 0.020412 | 0.030136 |  |

**IMPORT LOW: Simultaneous estimation of baseline and treatment effect, with adjustment for double counting**

The parameters below correspond to models 3 (exponential) and 4 (log normal)

Table 12

| Log scale - Model fitted on full sample (n = 1343) | | | | | | | | |
| --- | --- | --- | --- | --- | --- | --- | --- | --- |
| Model | Constant | |  | Treatment | |  | Sigma | |
|  | Coeff | SE |  | Coeff | SE |  | Coeff | SE |
| Exponential | -6.0686 | 0.3333 |  | -0.1220 | 0.4859 |  | - | - |
| Log-normal | 6.9856 | 1.1792 |  | 0.0761 | 0.4381 |  | 0.8542 | 0.2146 |

Table 13

| Exponential variance-covariance matrix | | |  | Log-normal variance-covariance matrix | | |
| --- | --- | --- | --- | --- | --- | --- |
|  | Treatment | Constant |  | Treatment | Constant | Sigma |
| Treatment | 0.2361 |  |  | 0.1919018 |  |  |
| Constant | -0.111111 | 0.111111 |  | -0.0703465 | 1.39061 |  |
| Sigma | - | - |  | 0.0041902 | 0.24454 | 0.04607 |

**FAST Forward: Simultaneous estimation of baseline and treatment effect, with adjustment for double counting**

The parameters below correspond to models 3 (exponential) and 4 (log normal)

Table 14

| Log scale - Model fitted on full sample (n = 2723) | | | | | | | | | | | |
| --- | --- | --- | --- | --- | --- | --- | --- | --- | --- | --- | --- |
| Model | Constant | |  | Subgroup | |  | Treatment | |  | Sigma | |
|  | Coeff | SE |  | Coeff | SE |  | Coeff | SE |  | Coeff | SE |
| Exponential | -5.0026 | 0.1837 |  | -0.6784 | 0.2827 |  | -0.6106 | 0.2719 |  | - | - |
| Log-normal | 5.7267 | 0.5110 |  | 0.7109 | 0.2649 |  | 0.4814 | 0.2533 |  | 0.8032 | 0.1141 |

Table 15

| Exponential variance-covariance matrix | | | |  | Log-normal variance-covariance matrix | | | |
| --- | --- | --- | --- | --- | --- | --- | --- | --- |
|  | Treatment | Subgroup | Constant |  | Treatment | Subgroup | Constant | Sigma |
| Treatment | 0.073935 |  |  |  | 0.064143 | - | - | - |
| Subgroup | 0.000016 | 0.079946 |  |  | 0.0038388 | 0.070187 | - | - |
| Constant | -0.02632 | -0.0244 | 0.03376 |  | 0.0030285 | 0.007884 | 0.2611 | - |
| Sigma | - | - | - |  | 0.0066437 | 0.007125 | 0.0546 | 0.01302 |

### Distant recurrence

#### Summary of point estimates

The table below summarises the point estimates for the treatment effects in each of the four models.

Table 16

| Distant recurrence: eligible to partial breast radiotherapy | | | | | | | | |
| --- | --- | --- | --- | --- | --- | --- | --- | --- |
| Model | |  | Relative treatment effect: 5F vs 15F (source: FF) | |  | Relative treatment effect: partial vs whole (source: IL) | |  |
|  |  |  | Hazard ratio | Acceleration factor |  | Hazard ratio | Acceleration factor |  |
|  |  |  | Coeff | Coeff |  | Coeff | Coeff |  |
| 1 | Exponential model, fit to control arms + HR from clinical trial |  | 1.27 | - |  | 0.88 | - |  |
| 2 | Exponential model, simultaneous estimation of baseline + HR |  | 1.28 | -0.24 |  | 0.92 | 0.08 |  |
| 3 | Exponential model, simultaneous estimation of baseline + HR with adjustment for double counting |  | 1.30 | -0.26 |  | 0.91 | 0.09 |  |
| 4 | Lognormal model, simultaneous estimation of baseline + AF with adjustment for double counting |  | - | -0.27 |  | - | 0.05 |  |

#### Parameters

**IMPORT LOW: Estimation of baseline only, no adjustment for double counting**

The parameters below correspond to the subgroup 1 baseline estimates for model 1

Log scale - Model fitted on control group (WB15F) only (n = 674).

Table 17

| Model | Constant | |  |
| --- | --- | --- | --- |
|  | Coeff | SE |  |
| Exponential | -5.7031 | 0.2774 |  |

**FAST Forward: Estimation of baseline only, no adjustment for double counting**

The parameters below correspond to the subgroup 2 baseline estimates for model 1

Log scale - Model fitted on partial breast eligible section of control group (WB15F) only (n = 753).

Table 18

| Model | Constant | |  |
| --- | --- | --- | --- |
|  | Coeff | SE |  |
| Exponential | -4.3259 | 0.1387 |  |

**IMPORT LOW: Simultaneous estimation of baseline and treatment effect, with no adjustment for double counting**

The parameters below correspond to model 2

Log scale - Model fitted on full sample (n = 1343).

Table 19

| Model | Constant | |  | Treatment | |  |
| --- | --- | --- | --- | --- | --- | --- |
|  | Coeff | SE |  | Coeff | SE |  |
| Exponential | -5.7031 | 0.2774 |  | -0.0830 | 0.4003 |  |

Table 20

| Exponential variance-covariance matrix | | |  |
| --- | --- | --- | --- |
|  | Treatment | Constant |  |
| Treatment | 0.1603 |  |  |
| Constant | -0.076923 | 0.076923 |  |
| Sigma | - | - |  |

**FAST Forward: Simultaneous estimation of baseline and treatment effect, with no adjustment for double counting**

The parameters below correspond to model 2

Log scale - Model fitted on full sample (n = 2723).

Table 21

| Model | Constant | |  | Subgroup | |  | Treatment | |  |
| --- | --- | --- | --- | --- | --- | --- | --- | --- | --- |
|  | Coeff | SE |  | Coeff | SE |  | Coeff | SE |  |
| Exponential | -4.3506 | 0.1347 |  | -1.6704 | 0.2475 |  | 0.2429 | 0.1735 |  |

Table 22

| Exponential variance-covariance matrix | | | |  |
| --- | --- | --- | --- | --- |
|  | Treatment | Subgroup | Constant |  |
| Treatment | 0.0301071 |  |  |  |
| Subgroup | -0.00007 | 0.061252 |  |  |
| Constant | -0.01694 | -0.008583 | 0.018152 |  |

**IMPORT LOW: Simultaneous estimation of baseline and treatment effect, with adjustment for double counting**

The parameters below correspond to models 3 (exponential) and 4 (log normal)

Log scale - Model fitted on full sample (n = 1343)

Table 23

| Model | Constant | |  | Treatment | |  | Sigma | |
| --- | --- | --- | --- | --- | --- | --- | --- | --- |
|  | Coeff | SE |  | Coeff | SE |  | Coeff | SE |
| Exponential | -5.7817 | 0.2887 |  | -0.0913 | 0.4174 |  | - | - |
| Log-normal | 7.2347 | 1.0749 |  | 0.0527 | 0.4316 |  | 0.9534 | 0.1847 |

Table 24

| Exponential variance-covariance matrix | | |  | Log-normal variance-covariance matrix | | |
| --- | --- | --- | --- | --- | --- | --- |
|  | Treatment | Constant |  | Treatment | Constant | Sigma |
| Treatment | 0.1742 |  |  | 0.1863023 |  |  |
| Constant | -0.083333 | 0.083333 |  | -0.0768756 | 1.15531 |  |
| Sigma | - | - |  | 0.0025438 | 0.19057 | 0.03413 |

**FAST Forward: Simultaneous estimation of baseline and treatment effect, with adjustment for double counting**

The parameters below correspond to models 3 (exponential) and 4 (log normal)

Log scale - Model fitted on full sample (n = 2723)

Table 25

| Model | Constant | |  | Subgroup | |  | Treatment | |  | Sigma | |
| --- | --- | --- | --- | --- | --- | --- | --- | --- | --- | --- | --- |
|  | Coeff | SE |  | Coeff | SE |  | Coeff | SE |  | Coeff | SE |
| Exponential | -4.4300 | 0.1398 |  | -1.6004 | 0.2488 |  | 0.2595 | 0.1791 |  | - | - |
| Log-normal | 5.0030 | 0.3101 |  | 1.5875 | 0.2485 |  | -0.2730 | 0.1862 |  | 0.7769 | 0.0775 |

Table 26

| Exponential variance-covariance matrix | | | |  | Log-normal variance-covariance matrix | | | |
| --- | --- | --- | --- | --- | --- | --- | --- | --- |
|  | Treatment | Subgroup | Constant |  | Treatment | Subgroup | Constant | Sigma |
| Treatment | 0.032071 |  |  |  | 0.034668 |  |  |  |
| Subgroup | -0.00008 | 0.061891 |  |  | -0.00332 | 0.061755 |  |  |
| Constant | -0.01817 | -0.00922 | 0.019554 |  | -0.0242 | 0.019531 | 0.096166 |  |
| Sigma | - | - | - |  | -0.00162 | 0.008413 | 0.021288 | 0.006011 |

## Estimate costs of health states

### Summary of trial data collected on resource use

**Resource use questions asked**

FAST Forward

1. How many times have you been visited by your GP for any reason (even if not related to your breast cancer)?
2. How many times have you visited your GP for any reason (even if not related to your breast cancer)?
3. How many times have you been visited by a district nurse?
4. How many times have you been visited by a MacMillan nurse?
5. How many days have you spent in hospital related to your breast cancer?
6. How many days have you spent in hospital for other reasons?
7. How many hospital outpatient visits have you had related to breast cancer?
8. How many hospital outpatient visits have you had for other reasons?

IMPORT LOW

1. How many times have you been visited by your GP for any reason (even if not related to your breast cancer)?
2. How many times have you visited your GP for any reason (even if not related to your breast cancer)?
3. How many times have you been visited by a district nurse?
4. How many times have you been visited by a MacMillan nurse?

Note that the questionnaires are identical except that questions 5-8 (i.e. those relating to inpatient and outpatient visits were not asked during IMPORT LOW). Because questions 5-8 have important resource implications, this means that the IMPORT LOW questionnaire is incomplete and so is excluded from further consideration.

**Resource use questionnaire data collection and recall period**

FAST Forward

Table 27

| Data collection timepoint | 0 months  (baseline) | 3 months | 6 months | 12 months | 24 months | 5 years |
| --- | --- | --- | --- | --- | --- | --- |
| Recall time | 3 months | 3 months* | 3 months | 6 months | 6 months | 6 months |

* Questions 5 to 8 (inpatient and outpatient visits) are qualitative “yes” “no” for the 3-month questionnaire. Without further assumptions and/or expert elicitation this would introduce a bias in the data. Therefore, 3-month data is excluded from further consideration.

### Attach cost data to resource use

The values described below were attached to the resource use questionnaire.

Table 28

| **Resource use questionnaire** | | | |
| --- | --- | --- | --- |
| **Question** | **Unit cost** | **Explanation** | **Source** |
| How many times have you been visited by your GP for any reason? | £167.99 | GP visit unit cost is £134.89 which includes travel time. Curtis et al., 2010 (PSSRU) used as not reported in more recent publications. Add to this average prescription cost per consultation £33.10 (Curtis et al., 2019). | [6,7] |
| How many times have you visited your GP for any reason? | £72.10 | GP surgery consultation unit cost £39 plus average prescription costs per visit £33.10. | [6] |
| How many times have you been visited by a district nurse? | £32.50 | District Nurse cost £44.22 per hour according to Curtis et al., 2018 (PSSRU) (inflated), Curtis et al., 2010 (PSSRU) reports face to face home visit for community nurse (includes district nurse) takes 20 minutes. Ratio of indirect to direct time for home visits is reported to be 1:1.21 resulting in total time 44.2 mins and cost £30.76 (this includes travel time). Curtis et al., 2010 (PSSRU) also reported travel costs of £1.74 (inflated) per visit. | [8,7] |
| How many times have you been visited by a MacMillan nurse? | £23.10 | Nurses cost £29 per hour according to MacMillan (2019) costing document. Curtis et al., 2010 (PSSRU) reports face to face home visit for community nurse takes 20 minutes. Ratio of indirect to direct time for home visits is reported to be 1:1.21 resulting in total time 44.2 mins and cost £21.36 (this includes travel time). Curtis et al., 2010 (PSSRU) also reported travel costs of £1.74 (inflated) per visit. | [7,9] |
| How many days have you spent in hospital related to your breast cancer? | £378.90 | Activity weighted average of elective and non-elective cost per day for Malignant Breast Disorders with and without Interventions reported in 2018/19 reference costs. Includes Elective Inpatients, Non Elective Inpatients,  Non-Elective Short Stay, Day Case and Regular Day or Night Admissions. | [10] |
| How many days have you spent in hospital for other reasons? | £568.88 | Activity weighted average of elective and non-elective inpatient costs per day reported in 2018/19 reference costs. | [10] |
| How many hospital outpatient visits have you had related to breast cancer? | £94.93 | Activity weighted average of outpatient procedure costs in clinical oncology (previously radiotherapy) reported in 2018/19 reference costs. | [10] |
| How many hospital outpatient visits have you had for other reasons? | £148.00 | Weighted average of cost for all outpatient attendances reported in 2018/19 reference costs. | [10] |

As shown previously, the questionnaire recall period differs across time periods, with the 6-month data collection period having a recall period of 3 months and data collection at 1 year, 2 years and 5 years having a 6 month recall period. To address this in the analysis, the total costs in the 6-month recall periods (1,2,5 year) are divided by 2. This results in all time periods estimating total costs over a 3-month period. This assumes that costs are constant over the recall period. These 3-month costs will then be multiplied by 4 to calculate the yearly costs required by the economic model.

### Classification of resource use observations into relevant health states

Analogous to the EQ-5D analysis, the aim here is to use the resource use questionnaire to estimate the average healthcare costs associated with the alive and disease-free state.

**Step 1: define valid resource use observations**

**Step 2: classify observations for the alive and disease-free health state**

**Step 3: calculate % missing resource use data by disease state**

For each time point in the alive and disease state calculate the number (and %) of missing resource use observations. This analysis will show the degree of missing data for the alive and disease-free health state.

**Missing data patterns for the alive and disease free health state**

The table below shows the number of observations and missing data patterns for the alive and disease-free state for the 6 month, 1 year, 2 year and 5 year data collection points.

Table 29

|  | Health state | | | | |  |  |
| --- | --- | --- | --- | --- | --- | --- | --- |
|  | Alive and disease free | | | |  | Death |  |
|  | Pat | Missing | | Mean |  | Pat |  |
| Month 6 | 1,163 | 128 | (11.0%) | £556 |  | 11 |  |
| Year 1 | 1,158 | 128 | (11.1%) | £340 |  | 14 |  |
| Year 2 | 1,129 | 157 | (13.9%) | £329 |  | 28 |  |
| Year 5 | 1,069 | 248 | (23.2%) | £295 |  | 64 |  |
| Pat=number of patients in health state (including missing), Missing=number and proportion of observations with missing 3 month costs in health state, Mean=average 3 month costs | | | | | | | |

**Descriptive statistics for alive and disease-free health state**

Table 30

| Variable | Alive and disease free | | | | |  |
| --- | --- | --- | --- | --- | --- | --- |
|  | Obs | Mean | Std Err | Min | Max |  |
| 3 month total costs |  |  |  |  |  |  |
| Month 6 | 1,035 | £556 | £1,293 | £0 | £24,255 |  |
| Year 1 | 1,030 | £340 | £445 | £0 | £4,682 |  |
| Year 2 | 972 | £329 | £638 | £0 | £13,720 |  |
| Year 5 | 821 | £295 | £533 | £0 | £7,991 |  |
| Std Err=standard error. All results pertain to average costs over a 3 month period. | | | | | | |

Figure 1 shows the average costs in the alive and disease-free state for previous 3 months over time.

Figure 1

This shows a clear discontinuity between the first observation (at 6 months) and the remaining observations.

### Estimating models

#### Choosing models for panel data analysis

The xtgee package in Stata was used to fit a generalised estimating equation (GEE) models to the 3 month cost data. An exchangeable within-individual correlation structure. A gamma family distribution was chosen to reflect the non-negativity and skewed distribution of cost data. A log link was chosen as this is a common choice for costs. An exchangeable correlation structure was utilised here.

The GEE model utilises all available observations of the dependent variable. This is equivalent to a complete case analysis (CCA). CCA was chosen as the degree of missing data in the study was low, less than 25% at any timepoint for both studies. This implies a missing completely at random assumption (MCAR) i.e. missingness is independent of both observed and unobserved values (Faria et al., 2014) [4].

#### FAST Forward

Data was restricted to only those known to be in the alive and disease-free state (see previous section for more details). When fitting the models, baseline costs were discarded as it is the post treatment costs that are to be estimated.

**Model with time discontinuity dummy and subgroup effect**

**Figure 1** shows that there is a large drop between costs in the first 6 months after treatment and the costs from 6 months onward. This discontinuity was modelled by defining a binary covariate indicating 6 months pre and post treatment. Eligibility for partial breast radiotherapy was also included in the analysis. Age and treatment were found not to be statistically significant and so were not included in the model.

For the group eligible for partial breast (subgroup 1) the mean costs over 3 months are estimated to be £304, with SE = £20.5. For the group ineligible for partial breast (subgroup 2) the mean costs over 3 months are estimated to be £353 with SE = £17. The mean costs over 3 months in the 1^st^ 6 months of the trial are £201 higher than in the remaining periods with a standard error of £32 (p = 0.000)

#### Results for use in economic model

The results above estimate average cots over a 3-month period. The economic model requires an estimate of average yearly costs.

**Yearly costs in subgroup 1: eligible for partial breast RT**

12 months are spent with an average cost of (4 x £304) = £1216, with SE* (4 x £20.5) = £82.

**Yearly costs in subgroup 2: ineligible for partial breast RT**

12 months are spent with an average cost of (4 x £353) = £1412, with SE* (4 x £17) = £68.

**Additional costs associated with first year after treatment**

In the 1^st^ year, the first 6 months will be spent with an additional cost of (2 x £201 =) £402 with SE* (2 x £32 =) £64. There are not additional costs in the second 6 months.

*For a random variable (X) with mean and variance, Var[c*X]= c^2⋅Var[X], where c is a constant. Therefore SE[c*X] = c*SE[X]

‘For a two normal distributions X and Y, the E(X + Y) = E(X) + E(Y) and Var(X + Y) = Var(X) + Var(Y). Therefore, SE(X + Y) = SE(X) + SE(Y).

# References

1. Woods BS, Sideris E, Sydes MR, Gannon MR, Parmar MK, Alzouebi M, Attard G, Birtle AJ, Brock S, Cathomas R (2018) Addition of docetaxel to first-line long-term hormone therapy in prostate cancer (STAMPEDE): Modelling to estimate long-term survival, quality-adjusted survival, and cost-effectiveness. European urology oncology 1 (6):449-458

2. Van Hout B, Janssen M, Feng Y-S, Kohlmann T, Busschbach J, Golicki D, Lloyd A, Scalone L, Kind P, Pickard AS (2012) Interim scoring for the EQ-5D-5L: mapping the EQ-5D-5L to EQ-5D-3L value sets. Value in health 15 (5):708-715

3. Ramos-Goñi JM, Ramallo-Fariña Y (2016) Eq5dds: A Command to Analyze the Descriptive System of EQ-5D Quality-of-life Instrument. The Stata Journal 16 (3):691-701. doi:10.1177/1536867x1601600309

4. Faria R, Gomes M, Epstein D, White IR (2014) A Guide to Handling Missing Data in Cost-Effectiveness Analysis Conducted Within Randomised Controlled Trials. PharmacoEconomics 32 (12):1157-1170. doi:10.1007/s40273-014-0193-3

5. Latimer N (2011) NICE DSU technical support document 14: survival analysis for economic evaluations alongside clinical trials-extrapolation with patient-level data. Sheffield: Report by the Decision Support Unit 2013

6. Curtis L, Burns A (2019) Unit costs of health and social care 2019. University of Kent, personal social services research unit, Cantebury

7. Curtis L, Netten A (2010) Unit costs of health and social care 2010. University of Kent, personal social services research unit, Canterbury

8. Curtis L, Burns A (2018) Unit costs of health and social care 2018. University of Kent, personal social services research unit, Canterbury

9. Macmillan (2019) The cost of Macmillan’s services fact sheet 2018. Macmillan Cancer Support,

10. England N (2020) 2018/19 National Cost Collection data. NHS England and NHS Improvement, London
